# Antioxidant and anti-apoptotic effects of curcumin-loaded nanoliposomes against di-n-butyl phthalate-induced hepatorenal damage in rats

**DOI:** 10.1007/s00210-026-05212-0

**Published:** 2026-04-07

**Authors:** Manal R. Bakeer, Maha M. Rashad, Omaima Ahmed, Fady Sayed Youssef, Ghada E. Ali, Seham Samir Soliman

**Affiliations:** 1 Department of Physiology, Faculty of Veterinary Medicine, Cairo University, Giza, 12211 Egypt; 2 Department of Biochemistry and Molecular Biology, Faculty of Veterinary Medicine, Cairo University, Giza, 12211 Egypt; 3 Department of Cytology and Histology, Faculty of Veterinary Medicine, Cairo University, Giza, 12211 Egypt; 4 Department of Pharmacology, Faculty of Veterinary Medicine, Cairo University, Giza, 12211 Egypt; 5 Department of Animal Reproduction and Artificial Insemination, Veterinary Research Institute, National Research Centre (NRC), Doki, Giza, 12622 Egypt

**Keywords:** Dibutyl phthalate, Curcumin, Nanoliposomes, Oxidative stress, Apoptosis, Hepatorenal toxicity

## Abstract

Di-n-butyl phthalate (DBP) is a conventional plasticizer known to induce hepatotoxicity and nephrotoxicity through oxidative stress and apoptosis. In contrast, curcumin, a polyphenolic compound from *Curcuma longa*, has significant antioxidant and anti-apoptotic activity; however, its therapeutic efficiency is limited due to poor absorption and low bioavailability. Nanoliposomes can significantly enhance the stability and absorption of curcumin, thereby improving its protective efficacy. Therefore, this study aimed to evaluate the protective effects of curcumin-loaded nanoliposomes against DBP-induced hepatotoxicity and nephrotoxicity in rats. The adult male rats were randomly assigned to four groups: control, CUR-NLs (200 mg/kg/day), DBP (500 mg/kg/day), and DBP + CUR-NLs, for 60 days. DBP administration significantly raised blood levels of lactate dehydrogenase (LDH), aspartate aminotransferase (AST), alanine aminotransferase (ALT), creatinine, uric acid, urea, and malondialdehyde (MDA), while lowering serum protein fractions and catalase (CAT) activity. Gene expression analysis revealed that nuclear factor erythroid 2-related factor 2 (*Nrf2*), Catalase (CAT), superoxide dismutase (SOD), and B-cell lymphoma-2 (BCL2) were downregulated, whereas cytochrome c (CYCS) and Bcl-2-associated X (Bax) were upregulated, and caspase-3 immunoreactivity increased. Compared with DBP-treated rats, co-treatment with CUR-NLs significantly restored protein metabolism, decreased lipid peroxidation, increased antioxidant defenses, raised anti-apoptotic markers, and improved histopathological and immunohistochemical findings. These results indicate that CUR-NLs effectively mitigate DBP-induced hepatorenal injury by restoring redox homeostasis and regulating apoptotic signaling pathways, suggesting that CUR-NLs represent a promising nanotechnological strategy for preventing DBP-induced organ toxicity.

## Introduction

DBP is one of the most commonly used plasticizers and is present in numerous consumer products, food packaging materials, and health care products, leading to its widespread presence in the environment as well as increased exposure to the human population (Bakeer et al. 2025a). Recent studies have demonstrated that DBP can lead to the induction of oxidative stress, mitochondrial damage, and apoptosis, ultimately resulting in hepatorenal toxicity in animal models and human populations (Zhao et al. 2016; Aly et al. 2021; Ibrahim et al. 2025a; Ivelja et al. 2025). The liver and kidneys, which are the primary organs for detoxification and excretion, are particularly vulnerable to DBP-induced toxicity (Bakeer et al. 2025b). Chronic exposure to DBP may be associated with an increase in lipid peroxidation, the consumption of endogenous antioxidants, disruption of redox homeostasis, and the activation of apoptotic signaling cascades (Lotfy et al. 2020). These harmful effects highlight the urgent need to develop novel protective strategies against DBP-induced organ dysfunction. Curcumin is a polyphenolic compound isolated from the rhizome of *Curcuma longa* that possesses well-established antioxidant, anti-inflammatory, and anti-apoptotic properties (Bakeer and Ibrahim 2025). Curcumin has been shown to scavenge reactive oxygen species (ROS), increase cellular endogenous protective mechanisms, and modulate apoptosis-related pathways (Hewlings And Kalman 2017). However, its use has been hampered by low systemic bioavailability, poor aqueous solubility, and rapid metabolism (Anand et al. 2007). This has led to many attempts to improve its delivery and therapeutic use. Among these is its encapsulation in liposomes. This technology improves curcumin stability and cellular uptake while prevent cellular senescence and apoptosis in various cells (Moballegh Nasery et al. 2020; Bakeer et al. 2025d). Curcumin-enriched nanoliposomes have shown successful controlled drug delivery and biodistribution and seem promising in preventing oxidative and apoptotic damage caused by environmental toxic substances like DBP (Gera et al. 2017).

Previous studies have demonstrated that curcumin and curcumin-based nanocarrier formulations can counteract DBP-induced reproductive toxicity, mainly by reducing testicular oxidative stress, apoptosis, and steroidogenic impairments (Bakeer et al. 2025a). These findings indicate the promising role of nanocarrier-mediated curcumin delivery in counteracting DBP-induced cytotoxicity. Conversely, despite the established vulnerability of the liver and kidneys to DBP accumulation and subsequent biotransformation, very limited evidence exists regarding the protective effects of curcumin nanoliposome against DBP-induced hepatorenal toxicity. Furthermore, the role of redox regulation and apoptosis signaling pathways in the DBP-induced alterations in the tissues remains an uncharted area. In this regard, the present study aimed to investigate, for the first time, the hepatorenal protective potentials of curcumin nanoliposomes in the rat model administered with DBP through integrated biochemical, histopathology, immunohistochemistry, and gene expression analyses.

## Materials and methods

### Ethics and animals

Adult male Wistar rats (200–230 g) were procured and maintained with unlimited access to water and food (22 ±  2 °C, 12 h dark/light cycle).

The animal study was approved by Cairo University’s institutional Animal Care and Use Committee (IACUC), which approved the experimental protocol (approval # Vet-CU 03162023631).

#### Clinical trial number

Not applicable.

### Chemicals

Unless otherwise noted, Sigma-Aldrich (St. Louis, MO, USA) provided all of the analytical chemicals and reagents utilized in this investigation. Di-n-butyl phthalate (CAS No. 84–74-2; 1,2-benzenedicarboxylic acid dibutyl ester; purity: 99.8%; density: 1.04 g/mL) and curcumin (purity: > 99.5%) were used as the main experimental compounds.

### Formulation of curcumin-loaded nanoliposomes

Curcumin-loaded nanoliposomes were synthesized utilizing the thin-film hydration process with minor modifications, as explained by Bisht et al. (2007). Briefly, curcumin, phosphatidylcholine, and cholesterol were dissolved in a round-bottom flask containing a mixture of chloroform and methanol (2:1, v/v). The solvent was removed by rotary evaporation to create a thin lipid film at 40 °C. The resulting film was hydrated with phosphate-buffered saline at 60 °C under continuous agitation for 1 h to produce multilamellar vesicles (MLVs). The dispersion was then sonicated (30 s on/off cycles for 10 min) to obtain nanoliposomes with reduced particle size.

### Description of CUR-NLs

The zeta potential and particle size distribution of curcumin-loaded nanoliposomes (CUR-NLs) were determined using dynamic light scattering (DLS) using a Zetasizer Nano ZS (Malvern Instruments, UK). The encapsulation efficiency (EE%) was evaluated using ultracentrifugation at 20,000 rpm for 1 h at 4 °C. Transmission electron microscopy (TEM; 70 kV, Carl Zeiss, Germany) was used to visualize the nanoliposomes of 20 µL of CUR-NLs formulation applied to a copper grid coated with carbon, stained with phosphotungstic dye, and allowed to dry (Gamal et al. 2021).

The amount of unencapsulated (free) curcumin in the supernatant was determined using a UV–Vis spectrophotometer set to 425 nm. The EE% was computed with the following calculation:

$$EE\mathrm{\%}=\frac{(Total\;curcumin-Free\;curcumin)}{Total\;curcumin}\times100$$

All characterization methods were performed as described by Kakkar et al. (2011).

### Stability testing

The stability of CUR-NLs was assessed by storing the samples in amber glass vials at 4 °C and 25 °C for 30 days. Zeta potential, Particle size, and encapsulation efficiency (EE%) were measured on days 0, 7, 14, and 30. The chemical stability of curcumin was determined spectrophotometrically at 425 nm, as described by Chang et al. (2012).

### Experimental design and dosing

Rats were divided into four groups at random (*n* = 10 per group) and given the following oral (p.o.) treatment once daily for 60 consecutive days:

1. Control group: Received vehicle only.
2. CUR-NLs group: Received CUR-NLs (200 mg/kg/day) was based on previous studies demonstrating its protective efficacy without toxicity (Salehi et al. 2020)
3. DBP group: Received di-n-butyl phthalate (500 mg/kg/day) (Srivastava et al. 1990)
4. DBP + CUR-NLs group: Received DBP (500 mg/kg/day) first, followed by CUR-NLs (200 mg/kg/day), administered 1 h apart.

Throughout the 60-day experimental period, all animals were observed daily for general health and clinical signs of toxicity.

### Sample collection

At the end of the study, rats were euthanized using isoflurane anesthesia and cardiac exsanguination. Blood samples were collected from the femoral artery, and serum was separated by centrifugation at 4,500 × g for 10 min. To minimize enzymatic degradation, each tissue was homogenized (10% w/v) in ice-cold phosphate-buffered saline (PBS, pH 7.4) with a glass-Teflon homogenizer on ice. The homogenates were centrifuged at 10,000 × g for 15 min at 4°C. The supernatants were then collected for biochemical analysis. All tissue homogenates and serum samples were aliquoted and kept at −80°C until needed. Additional samples were preserved in Bouin’s Fixative for histological and immunohistochemical analysis (Mohamed et al. 2017; Abdoon et al. 2023).

### Biochemical assessments

Biochemical assays were performed with commercial kits obtained from Bio Diagnostic Co. (Giza, Egypt). The activities of alanine aminotransferase (ALT), aspartate aminotransferase (AST), and lactate dehydrogenase (LDH) were measured in accordance with Abdoon et al. (2020) and Ibrahim et al. (2025b). Renal function markers: serum creatinine, uric acid, and urea concentrations were determined in accordance with Bakeer et al. 2025e)**.** Oxidative stress indices: The levels of malondialdehyde (MDA) and catalase (CAT) activity were determined using the methods of Soliman et al. (2025a) and Ismael et al. (2025).

### Real-time quantitative PCR (qRT-PCR) analysis

The relative mRNA expression levels of *Nrf2*, CAT, SOD, CYCS, Bax, and BCL2 in hepatic and renal tissues were evaluated using real-time quantitative PCR (qRT-PCR), with GAPDH as the internal reference gene (Zein et al. 2026). Total RNA was extracted using Applied Biotechnology’s Total RNA Extraction Kit (EX02). RNA quality and concentration were spectrophotometrically determined, and complementary DNA (cDNA) was synthesized using the Applied Biotechnology cDNA Synthesis Kit (AMP11) (Bakeer et al. 2025e). SYBR Green Master Mix (Applied Biotechnology, AMP03) was used to perform quantitative PCR (Azouz et al. 2026). The primer sequences are shown in Table 1. Each run included negative controls that lacked the template to guarantee that there was no contamination (Elmosalamy et al. 2022). All reactions were performed in triplicate. The relative gene expression was estimated using the 2^ − ΔΔCT technique (Mohamed et al. 2024).

**Table 1:** The list of Primer sequences for quantitative real-time PCR

Gene symbol	Gene description	Primer Sequence	Reference
*GAPDH* *NC_005103.4*	Glyceraldehyde3-phosphate dehydrogenase	F:—5′-ACCACAGTCCATGCCATCAC-3′ R:—5′-TCCACCACCCTGTTGCTGTA-3′	Hashim et al. 2024
*Nrf 2* *NC_005102.4*	Nuclear factor, erythroid 2-like 2	F: −5′‐GGCCCTCAATAGTGCTCAG‐3′ R: −5′‐TAGGCACCTGTGGCAGATTC‐3′	Morgan et al. 2023
*CAT* *NM_012520.2*	Catalase	F: −5′‐ ACACTTTGACAGAGAGCGGA‐3′ R: −5′‐ TTTCACTGCAAACCCACGAG‐3′	Abdelghafar et al. 2025
*SOD* *NM_017050*	Superoxide dismutase	F: 5′‐ -GCAGAAGGCAAGCGGTGAAC‐3′ R:5′‐ TAGCAGGACAGCAGATGAGT-3′	Ahmed et al. 2024
*CYCS* *K00750.1*	Cytochrome C	F: −5′‐ TAC CC T CTC AAC GACAGC AG‐3′ R: −5′‐ TCT TGA CAT TCT CCT CGG TG‐3′	Bashir et al. 2025
*Bax* *NM_017059.2*	BCL2-associated X	F: 5′‐ TCATGAAGACAGGGGCCTTT ‐3′ R:5′‐ CTGCAGCTCCATGTTGTTGT −3′	Noshy et al. 2023
*BCL2* *NM_016993.2*	B‐cell lymphoma 2	F: 5′‐ CTTCAGGGATGGGGTGAACT ‐3′ R:5′‐ ATCAAACAGAGGTCGCATGC −3′	Rashad et al. 2018

### Histopathology

The liver and kidney tissues were fixed in Bouin’s fixative, dehydrated with ethanol in a graded sequence, cleared with xylene, and embedded in paraffin wax. To evaluate histopathology, tissue slices (3–4 μm thick) were cut and stained with hematoxylin and eosin, as described by Bancroft and Gamble (2013).

### Immunohistochemistry of caspase-3

Caspase-3 immunohistochemistry was carried out on 5 μm paraffin-embedded tissue slices. Antigens were extracted for 15 min by heating in a microwave with 10 mM sodium citrate buffer (pH 6.0). To decrease endogenous peroxidase activity, methanol was combined with 0.3% hydrogen peroxide (H₂O₂) for 15 min at room temperature. The sections were then treated with a primary monoclonal anti-caspase-3 antibody overnight at 4 °C (Thermo Fisher Scientific, Cat. No. 43–7800; 1:100 dilution in PBS containing 1% BSA). After washing, the slides were coated with an HRP-conjugated secondary antibody, and the immunoreactivity was measured with a diaminobenzidine (DAB) substrate. The slices were counterstained with hematoxylin, dried, and mounted. The positive control was done by using lymph node tissue, while the negative control was done by omitting the primary antibody to ensure staining specificity. Positive caspase-3 immunostaining (brown cytoplasmic coloring) was examined under a light microscope.

Caspase-3 immunostaining in the liver and kidney tissue sections was quantified using a Leica Quin 500 image analyzer (Leica Microsystems, Switzerland). Five randomly selected fields per slide were analyzed at 400 × magnification, with five samples per group (*n* = 5 for every group). Five non-overlapping microscopic fields from each animal were examined and photographed under a light microscope. The results were presented as the percentage of the positively stained area representing caspase-3 expression (Abdoon et al. 2024).

### Statistical analysis

Data were expressed as mean ± SD. One-way ANOVA was performed to assess differences between groups, followed by Tukey’s post hoc test using SPSS Statistics version 27.0. A p-value of < 0.05 was considered statistically significant.

## Results

### Curcumin-loaded nanoliposomes characterization

**Fig. 1:**
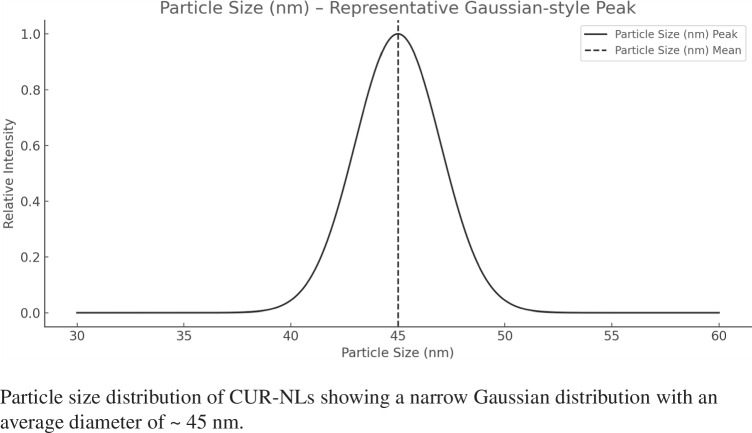
Particle size distribution of curcumin-loaded nanoliposomes measured by DLS

**Fig. 2:**
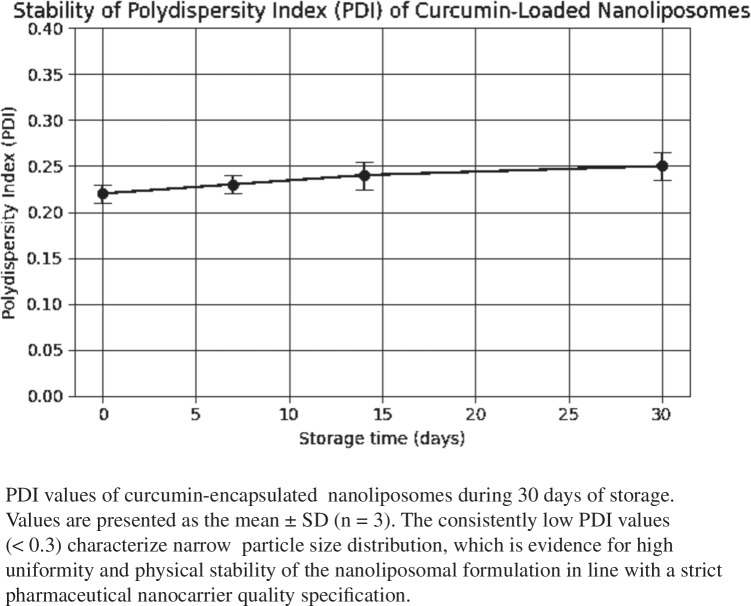
The Polydispersity Index (PDI) for Stability of Curcumin-Loaded Nanoliposomes

**Fig. 3:**
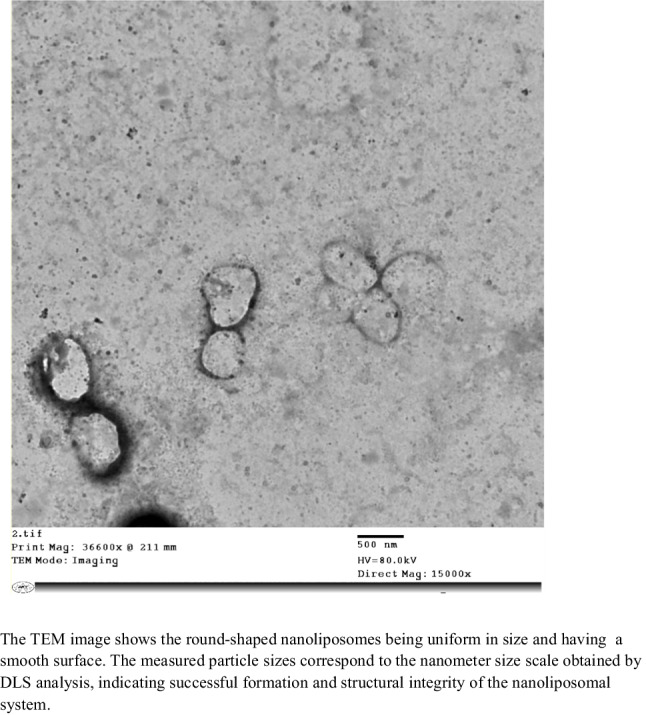
TEM image of Curcumin Loaded Nanoliposome

The physicochemical characterization of curcumin-loaded nanoliposomes demonstrated that the formulation was well-optimized and suitable for potential biomedical applications. The particle size distribution was quite uniform (Fig. 1), with an average diameter of approximately 45 nm**,** indicating successful fabrication of nanosized and monodisperse liposomes. Furthermore, the PDI values remained consistently below 0.3 throughout the storage period (Fig. 2). Morphological examination using transmission electron microscopy (TEM) further confirmed the formation of spherical and well-defined nanoliposomes (Fig. 3). The observed morphology was in good agreement with the DLS and PDI results, supporting the uniformity and physical stability of the prepared formulation.

**Fig. 4:**
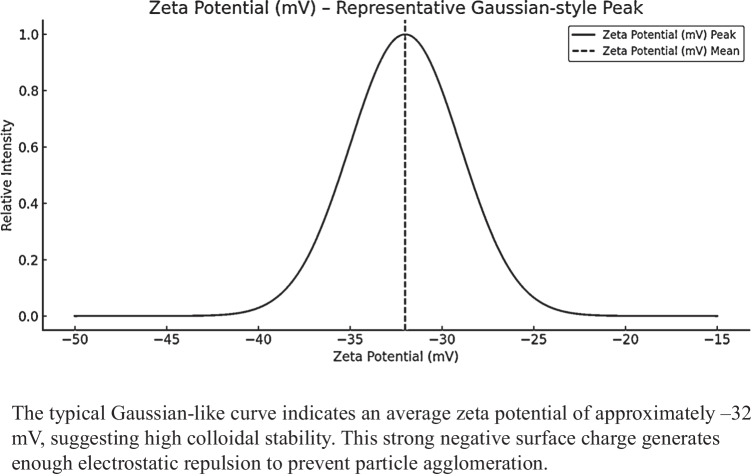
Zeta potential distribution of the prepared formulation showing surface charge characteristics and particle stability

**Fig. 5:**
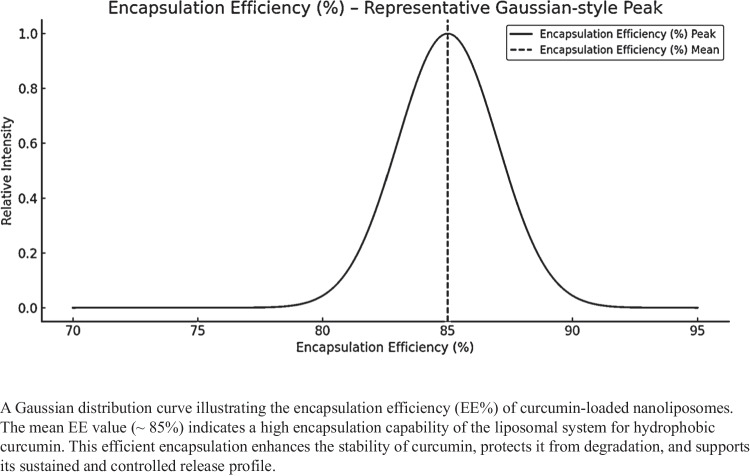
Encapsulation efficiency distribution of curcumin-loaded nanoliposomes

The zeta potential analysis (Fig. 4) showed a mean surface charge of –32 mV, suggesting excellent colloidal stability due to electrostatic repulsion that minimizes nanoparticle aggregation. This high stability helps maintain the structural integrity of the nanoliposomes during storage and systemic circulation. The encapsulation efficiency (EE%) was also high, averaging 85% (Fig. 5), reflecting the strong hydrophobic interaction between curcumin and the lipid bilayer. This efficient encapsulation promotes sustained release and protects curcumin from premature degradation.

**Fig. 6:**
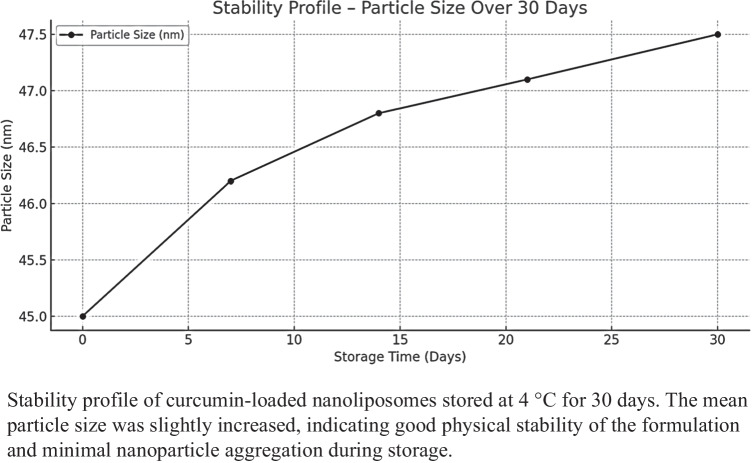
Particle Size Stability Over Time

**Fig. 7:**
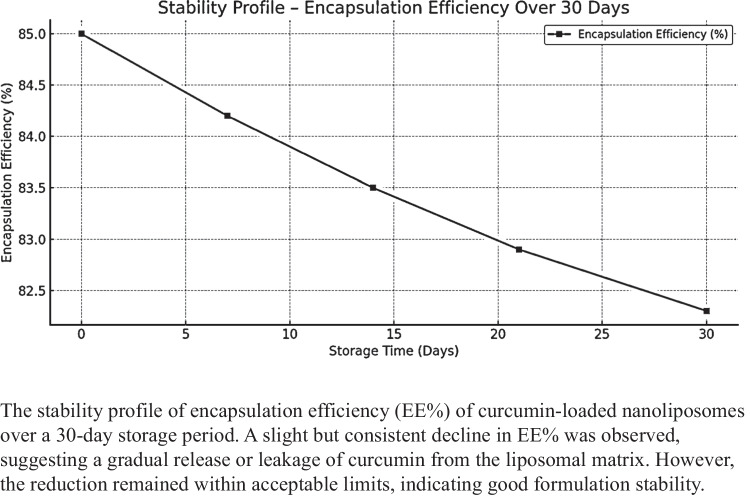
Stability of Encapsulation Efficiency (EE%) of Curcumin-Loaded Nanoliposomes Over Time

Storage stability studies further confirmed the robustness of the formulation. As shown in Fig. 6, the particle size exhibited negligible variation after 30 days of storage at 4 °C, indicating favorable physical stability. Similarly, Fig. 7 shows that only a minor reduction in EE% occurred during storage, suggesting that curcumin remained effectively retained within the liposomal matrix. Overall, these results confirm that the developed curcumin-loaded nanoliposomes possess desirable physicochemical properties, including nanoscale uniformity, high stability, and efficient encapsulation, making them a promising nanocarrier system for mitigating DBP-induced oxidative and histopathological damage.

### Effects of CUR-NLs on serum protein profile

Serum total protein, albumin, globulin concentrations, and albumin/globulin (A/G) ratio were significantly lower than those of the control group after DBP exposure, as shown in Table 2. Conversely, CUR-NLs alone significantly increased total protein and globulin levels, along with a moderate elevation in the A/G ratio, suggesting a stimulatory effect on hepatic protein synthesis and immunoglobulin production. Notably, co-administration of CUR-NLs with DBP partially restored total protein, globulin, and the A/G ratio toward normal values compared to those of the DBP-treated group, although values don’t completely return to control levels.

**Table 2:** Co-treatment with dibutyl phthalate and CUR-NLs affects serum total protein, albumin, globulin, and albumin/globulin ratio

Treatment/parameters	T. Proteins g/dl	Albumin g/dl	Globulin g/dl	Albumin/Globulin
Control	**6.5 ± 0.07 **^**c**^	** 2 ± 0.02 **^**b**^	**4.5 ± 0.09 **^**c**^	**0.45 ± 0.01 **^**b**^
CUR-NLs	**9.1 ± 0.06 **^**d**^	** 2 ± 0.01 **^**a,b**^	**7.1 ± 0.07 **^**d**^	**0.64 ± 0.01 **^**c**^
DBP	**4.8 ± 0.06 **^**a**^	**1.9 ± 0.01 **^**a**^	**2.8 ± 0.08 **^**a**^	**0.3 ± 0.01 **^**a**^
DBP + CUR-NLs	**6.1 ± 0.08 **^**b,c**^	** 2 ± 0.01 **^**a,b**^	**4.1 ± 0.07 **^**b.c**^	**0.49 ± 0.01 **^**b**^

Data are presented as (mean ± SD) in rats. Different superscript letters within the same column indicate significant differences among groups (*p* < 0.05), *n* = 10 rats per group. DBP, di-n-butyl phthalate; CUR-NLs, curcumin-loaded nanoliposomes.

### CUR-NLs prevent DBP-induced hepatic dysfunction

Administration of DBP led to a marked and significant increase in serum AST, ALT, and LDH activities compared to the control group, as demonstrated in Table 3. In contrast, CUR-NLs supplementation alone significantly decreased AST, ALT, and LDH activities below control values. Co-administration of CUR-NLs with DBP significantly attenuated the DBP-induced elevation of these enzymes, although their levels remained a little higher than those of the control group.

**Table 3:** Co-treatment with DBP and CUR-NLs on ALT, AST, and LDH levels

Treatment/parameters	AST U/L	ALT U/L	LDH U/L
Control	**3.6 ± 0.1 **^**b,c**^	** 4.9 ± 0.5 **^**a**^	** 70 ± 7 **^**b**^
CUR-NLs	**1.6 ± 0.2 **^**a**^	** 3.1 ± 0.2 **^**a**^	** 48.4 ± 2.3 **^**a**^
DBP	**13 ± 1.4 **^**d**^	**22.2 ± 1.4 **^**c**^	**146.9 ± 4.1 **^**d**^
DBP + CUR-NLs	**4.8 ± 0.2 **^**b,c**^	**13.7 ± 1 **^**b**^	**106.8 ± 2.2 **^**c**^

Data are presented as (mean ± SD) in rats. Different superscript letters within the same column indicate significant differences among groups (*p* < 0.05), *n* = 10 rats per group. DBP, di-n-butyl phthalate; CUR-NLs, curcumin-loaded nanoliposomes

### Curcumin improves renal function

Administration of DBP resulted in a significant promotion of serum creatinine, urea, and uric acid levels compared to the control, as shown in Table 4. Conversely, rats given CUR-NLs exhibited renal function parameters similar to those of the control group; CUR-NLs themselves did not adversely affect kidney function. Co-administration of curcumin with DBP markedly attenuated the DBP-induced increase in serum creatinine, urea, and uric acid levels, although the readings did not completely return to normal**.**

**Table 4:** Effect of co-treatment with di-n-butyl phthalate and CUR-NLs on serum creatinine, Uric Acid, and Urea concentration

Treatment/parameters	Creatinine (mg/dl)	Uric Acid (mg/dL)	Urea (mg/dL)
Control	**1.4 ± 0.02 **^**b**^	**2.55 ± 0.58 **^**a**^	**24.3 ± 5.1 **^**a**^
CUR-NLs	**1.3 ± 0.02 **^**a**^	**2.81 ± 0.75 **^**a**^	**23.65 ± 6.8 **^**a**^
DBP	**1.6 ± 0.01**^**d**^	**5.42 ± 0.81 **^**b**^	**55.2 ± 6.2 **^**b**^
DBP + CUR-NLs	**1.5 ± 0.01**^**c**^	**4.69 ± 0.92 **^**ab**^	**38.7 ± 2.1 **^**a**^

Data are presented as (mean ± SD) in rats. Different superscript letters within the same column indicate significant differences among groups (*p* < 0.05), *n* = 10 rats per group. DBP, di-n-butyl phthalate; CUR-NLs, curcumin-loaded nanoliposomes

### Oxidative stress and antioxidant defense

Exposure to DBP markedly elevated serum MDA levels while significantly reducing catalase activity compared with the control, as presented in Table 5. CUR-NLs supplementation alone significantly reduced MDA concentrations and markedly increased catalase activity. In the DBP + CUR-NLs-treated group, MDA levels were significantly lower and catalase activity significantly higher than those observed in the DBP group, although neither parameter completely returned to control values.

**Table 5:** Co-treatment with DBP and CUR-NLs effects on MDA levels and catalase activity

Treatment/parameters	MDA nmol/ml	Catalase U/mg
Control	**8.9 ± 0.2**^**b**^	**10.2 ± 0.1**^**b**^
CUR-NLs	**6.4 ± 0.1**^**a**^	**36.2 ± 0.6**^**d**^
DBP	** 11 ± 0.2**^**d**^	** 4 ± 0.1**^**a**^
DBP + CUR-NLs	**9.6 ± 0.2**^**c**^	**20.7 ± 2.6**^**c**^

Data are shown as (mean ± SD) in rats. Different superscript letters within the same column indicate significant differences among groups (*p* < 0.05), *n* = 10 rats per group. DBP, di-n-butyl phthalate; CUR-NLs, curcumin-loaded nanoliposomes

### Relative mRNA expression of antioxidant-related genes (*Nrf2*, CAT, and SOD)

**Fig. 8:**
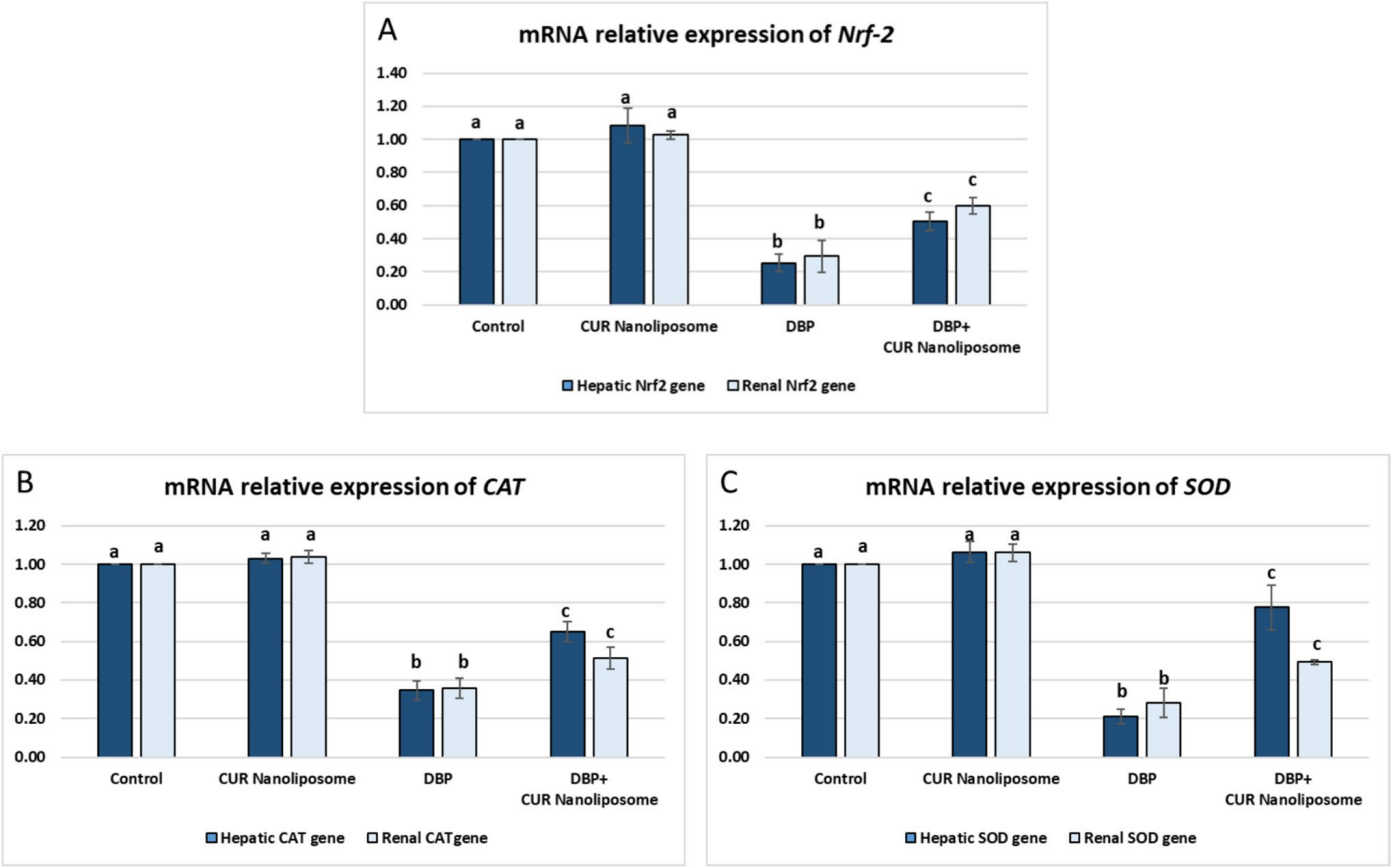
Effects of di-n-butyl phthalate (DBP) and/or curcumin-loaded nanoliposomes (CUR-NLs) on the relative mRNA expression levels of antioxidant-related genes in the liver and kidney tissues of rats: **A** Nuclear factor erythroid 2–related factor 2 (*Nrf2*), **B** catalase (CAT), and **C** superoxide dismutase (SOD). Data are presented as mean ± SE (*n* = 5 rats per group). Bars labeled with different letters differ significantly at *p* < 0.05 (ANOVA followed by Tukey’s test)

DBP exposure caused a significant downregulation of *Nrf2*, *CAT*, and *SOD* gene expression in both hepatic and renal tissues compared with the negative control group (*p* < 0.05). Conversely, co-treatment with curcumin-loaded nanoliposomes (CUR-NLs) significantly upregulated the expression of these genes in both tissues relative to the DBP-treated group (*p* < 0.05), as seen in Fig. 8.

### mRNA relative expression of apoptosis-related genes (CYCS, Bax, and BCL-2)

**Fig. 9:**
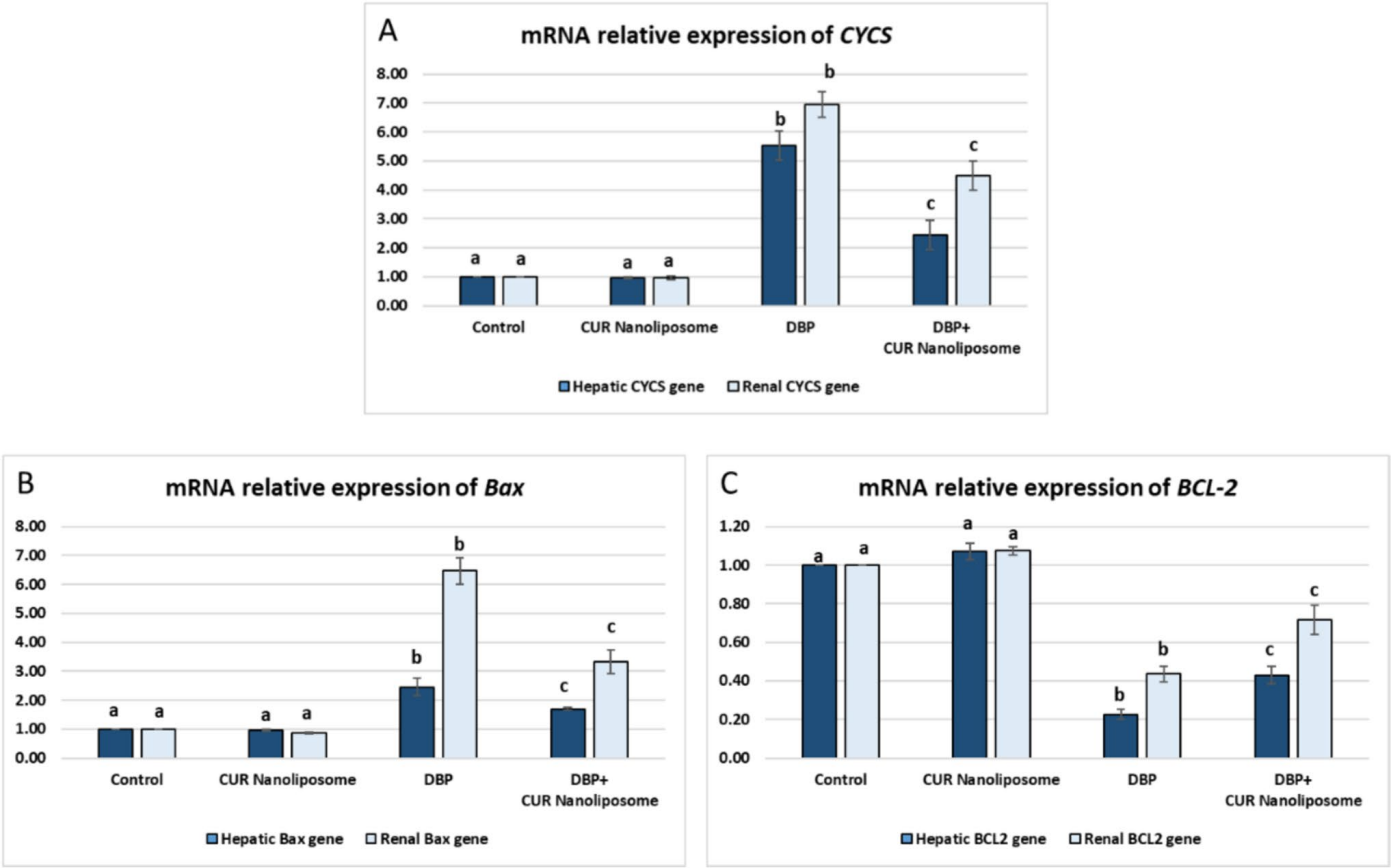
Effects of di-n-butyl phthalate (DBP) and/or curcumin-loaded nanoliposomes (CUR-NLs) on the relative mRNA expression of apoptosis-related genes in the hepatic and renal tissues of rats: **A** cytochrome c, somatic (CYCS), **B** BCL2-associated X protein (Bax), and **C** B-cell lymphoma 2 (BCL-2). Values are expressed as mean ± SE (*n* = 5 rats per group). Groups with different letters differ significantly at *p* < 0.05, whereas those sharing the same letter are not significantly different

As illustrated in Fig. 9, DBP exposure significantly upregulated the hepatic and renal expression levels of *CYCS* and *Bax* genes compared with the control group (*p* < 0.05), indicating activation of the apoptotic pathway. Co-treatment with curcumin-loaded nanoliposomes (CUR-NLs) significantly downregulated the expression of these pro-apoptotic genes relative to the DBP-treated group (*p* < 0.05). Conversely, *BCL-2* gene expression was markedly downregulated in the liver and kidney tissues of the DBP-treated group compared with the control group (*p* < 0.05). However, co-administration of CUR-NLs significantly upregulated *BCL-2* expression in both tissues compared with the DBP-treated group (*p* < 0.05), as shown in Fig. 9.

### Histopathological examination

#### Light microscopy observations

**Fig. 10:**
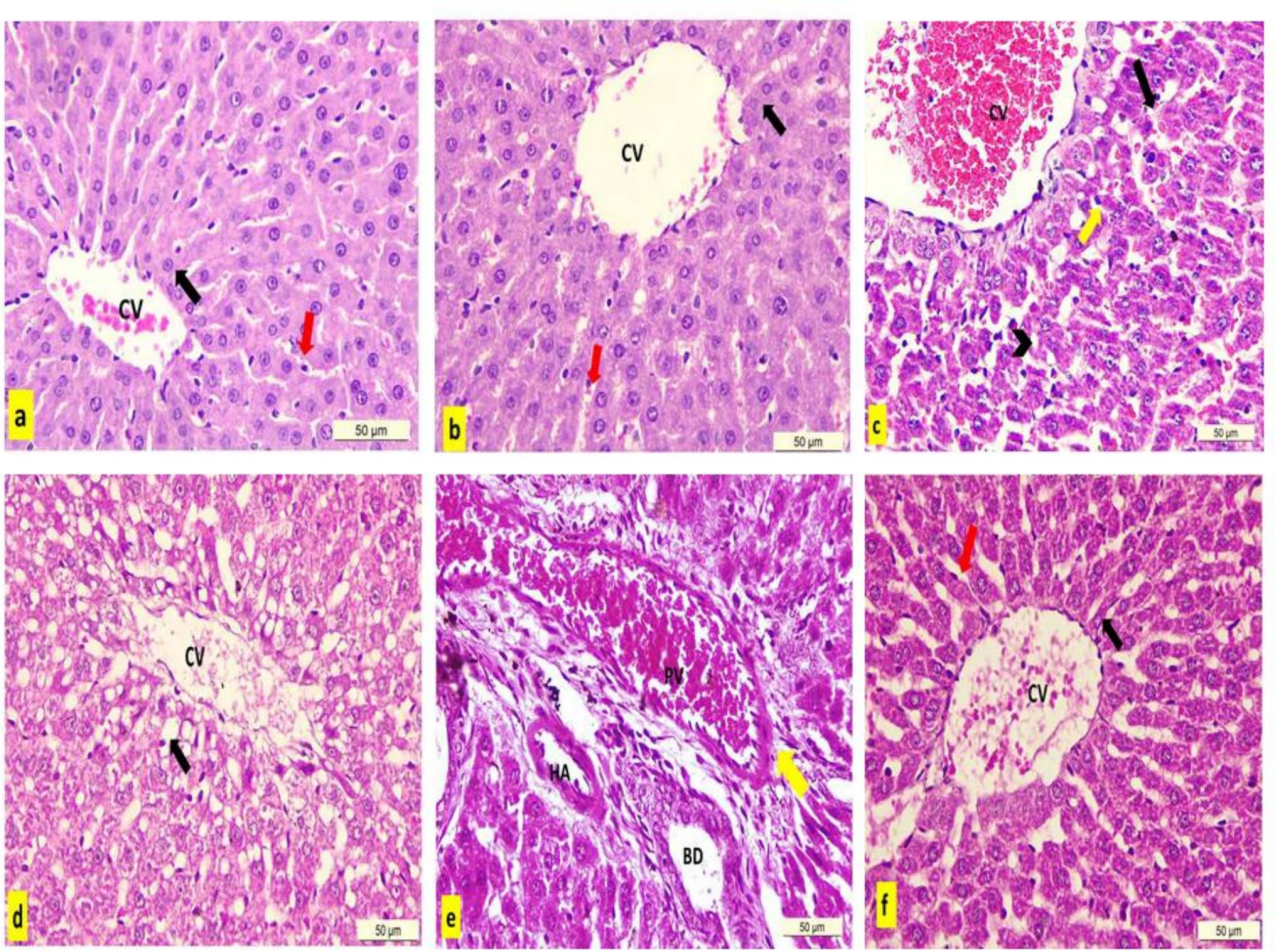
**a**–**f** Photomicrographs of liver sections from adult male albino rats (H&E, × 400). **a**, **b** Control and CUR-NLs -treated groups showing normal hepatic architecture with radiating hepatic cords (black arrows) extending from the central vein (CV) and separated by normal hepatic sinusoids (red arrows). **c**–**e** Liver sections from the DBP-exposed group showing marked histopathological alterations: **c** congested and dilated central vein (CV) and hepatic sinusoids (black arrows), disorganization of hepatic cords (black chevrons), and proliferation of Kupffer cells (yellow arrows); **d** degenerative changes in hepatocytes with cytoplasmic vacuolation (black arrows); **e** portal area showing a bile duct (BD) and a branch of the hepatic artery (HA) surrounded by inflammatory cell infiltration around the dilated and congested portal vein (PV). **f** Liver section from the DBP + CUR-NLs -treated group showing restoration of normal hepatic architecture with regularly arranged hepatic cords (black arrows) and normal hepatic sinusoids without congestion or dilatation (red arrows)

Histopathological analysis of liver tissue sections from adult male albino rats stained with hematoxylin and eosin revealed that both the control and CUR-NLs-treated groups exhibited normal hepatic architecture. The hepatic cords radiating from the central vein consisted of polygonal hepatocytes with central, spherical, and vesicular nuclei. These cords were separated by normal hepatic sinusoids (Fig. 10 a,b). In contrast, liver sections from the DBP-intoxicated group showed marked pathological alterations, including congestion and dilation of the central vein and hepatic sinusoids, disorganization of hepatic cords, and proliferation of Kupffer cells (Fig. 10c). The hepatocytes exhibited degenerative changes, such as cytoplasmic vacuolation and nuclear pyknosis (Fig. 10d). In addition, inflammatory cell infiltration was observed around the dilated and congested portal vein (Fig. 10 e). Treatment with CUR-NLs markedly alleviated DBP-induced hepatic damage, as evidenced by the restoration of normal hepatic architecture with hepatocytes radiating from the central vein and nearly normal blood sinusoids, although mild dilation of the central vein persisted (Fig. 10 f).

**Fig. 11:**
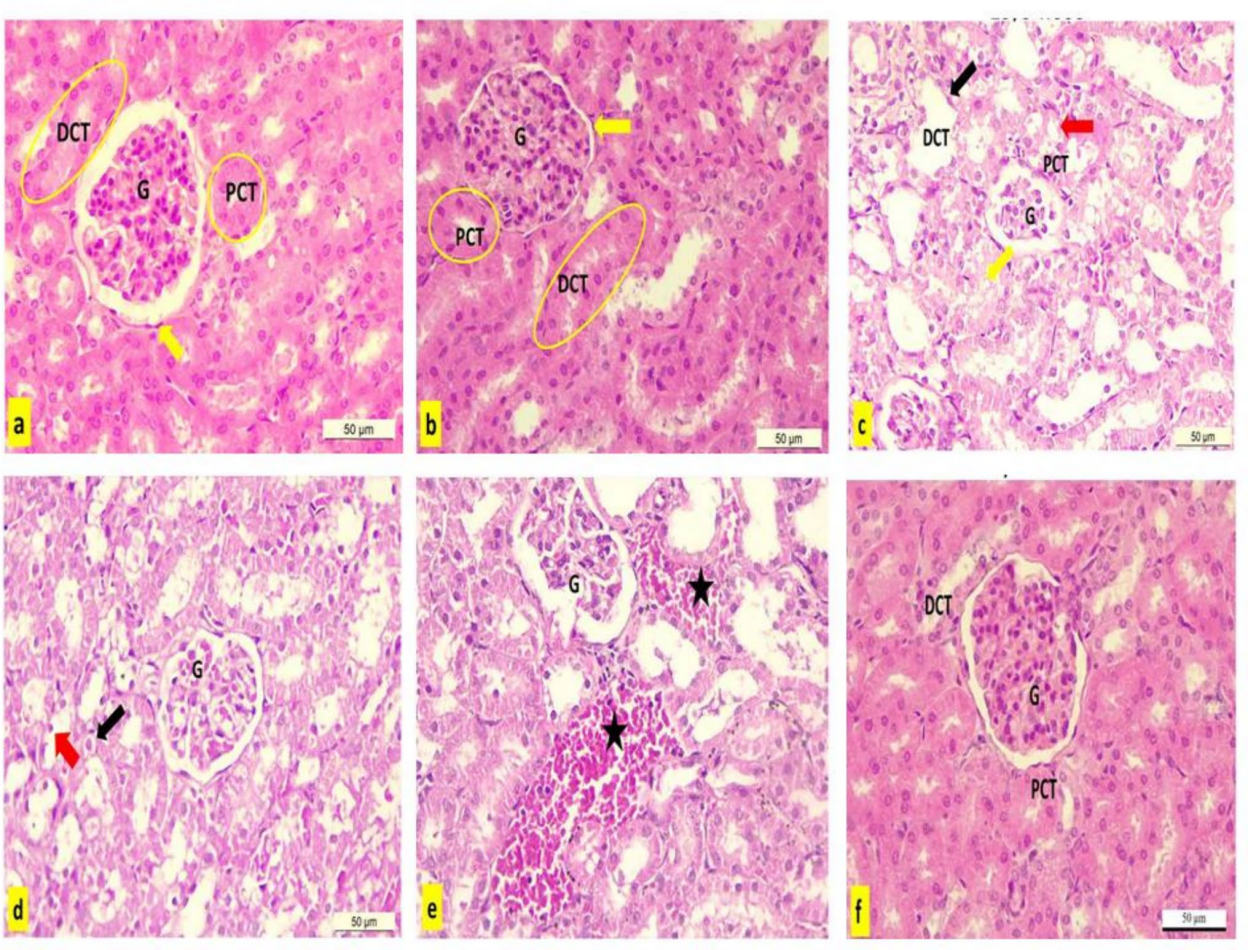
**a**–**f** Photomicrographs of renal cortex sections from adult male albino rats (H&E, × 400). **a** Control group and **b** CUR-NLs -treated group showing normal renal corpuscles with intact glomerular capillary tufts (G) and Bowman’s capsules (yellow arrow). The proximal convoluted tubules (PCT) display narrow lumina lined with large epithelial cells, while the distal convoluted tubules (DCT) exhibit wider lumina lined with cuboidal cells. **c**–**e** DBP-treated group showing: **c** Distorted and shrunken glomeruli (G), hydropic degeneration and vacuolization of PCT cells (red arrow), and DCTs lined by flattened (squamous) epithelial cells (black arrow); some tubules showed desquamation of their luminal contents (yellow arrow).**d** Congested glomerular capillary tufts (G) with DCT cells exhibiting vacuolization (black arrow) and nuclear pyknosis (red arrow). **e** Glomeruli with markedly widened Bowman’s spaces (G) and severe interstitial congestion (black star). **f** CUR-NLs + DBP co-treated group showing nearly normal renal cortical architecture with minimal degeneration and reduced vascular congestion

Histological examination of kidney sections from the control and CUR-NLs -treated groups of adult male albino rats, stained with hematoxylin and eosin (H&E), revealed a normal renal cortical architecture. The renal cortex consisted of renal corpuscles containing well-defined glomerular capillary tufts enclosed by intact Bowman’s capsules, proximal convoluted tubules (PCTs) with narrow lumina lined by large epithelial cells, and distal convoluted tubules (DCTs) with wider lumina lined by cuboidal epithelial cells (Fig. 11 a, b). In contrast, kidney sections from the DBP-intoxicated group exhibited marked histopathological alterations compared with the control and curcumin groups. The renal cortex showed distorted and shrunken glomeruli (Fig. 11 c); some glomeruli displayed capillary tuft congestion (Fig. 11 d), while others revealed widened Bowman’s spaces (Fig. 11 e). Additionally, hydropic degeneration and vacuolization were evident in PCT cells. Some DCT segments were lined by flattened squamous cells or showed sloughing of epithelial debris into the lumen (Fig. 11 c). Other DCT cells exhibited cytoplasmic vacuolization and nuclear pyknosis (Fig. 11 d). Severe interstitial vascular congestion was also observed (Fig. 11 e). In contrast, kidney tissues from the DBP + CUR-NLs-treated group showed a marked improvement in renal cortical structure, approaching normal histoarchitecture with minimal signs of degeneration or congestion (Fig. 11f).

**Fig. 12:**
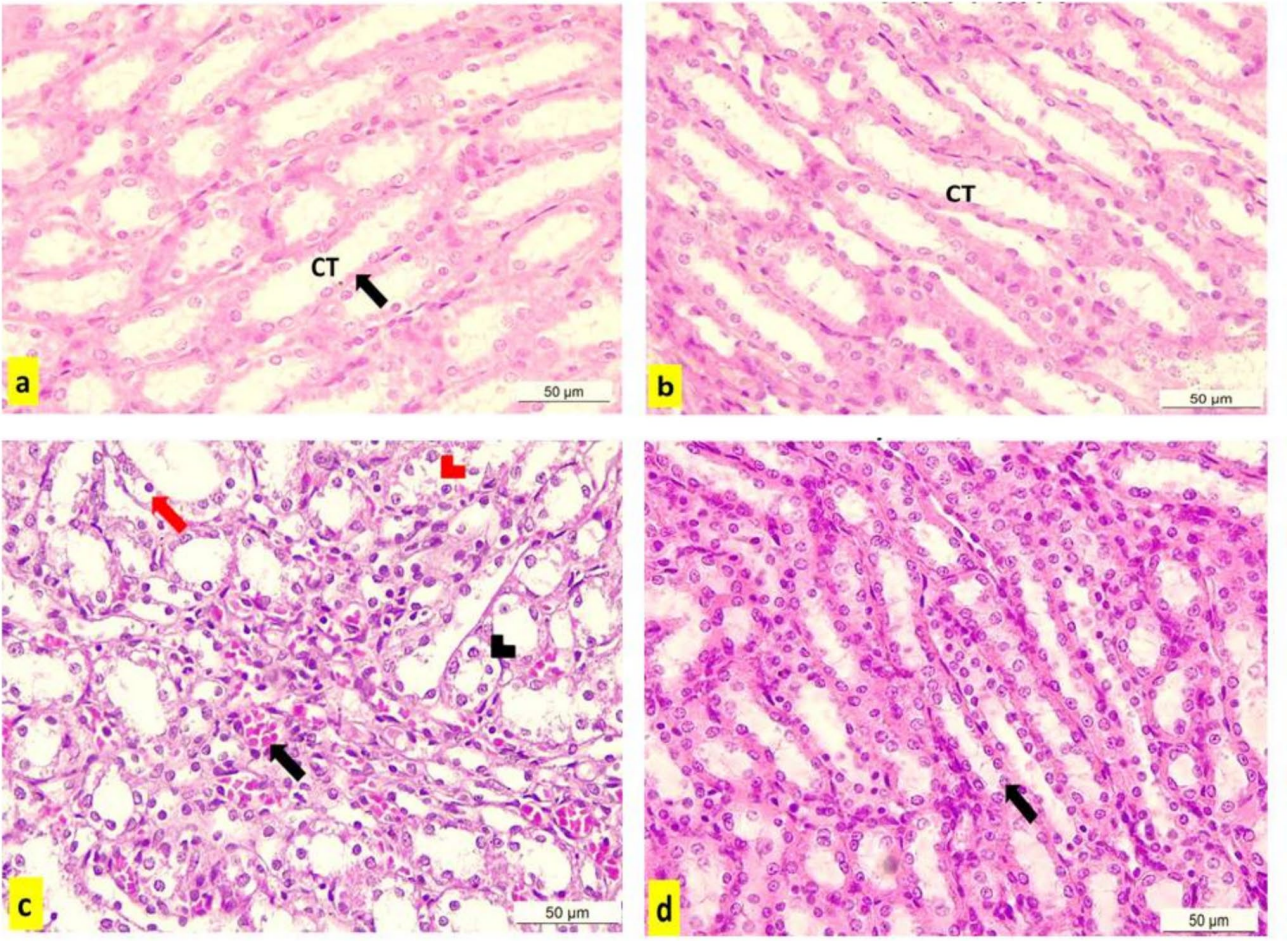
**a**–**f** Renal medulla sections from adult male albino rats (H&E, × 400). **a** Control and **b** CUR-NLs groups showing normal collecting tubules (CTs) lined with cuboidal epithelial cells (black arrows). **c** DBP-treated group showing vacuolar degeneration of the collecting tubules (red chevrons), loss of cytoplasmic eosinophilia in some tubular cells (black chevrons), and pyknotic nuclei (red arrows). Interstitial hemorrhage is also observed (black arrows). **d** DBP + CUR-NLs group showing collecting tubules lined with normal cuboidal cells having spherical nuclei (black arrows) and absence of interstitial hemorrhage

The renal medulla of both the control and CUR-NLs groups showed normal histological features, with collecting tubules lined by cuboidal epithelial cells (Fig. 12 a,b). In contrast, the DBP-exposed group exhibited marked degenerative changes in the collecting tubules, including vacuolar degeneration, loss of cytoplasmic acidophilia, and pyknotic nuclei in some cells, along with interstitial hemorrhage (Fig. 12 c). However, the curcumin-treated group displayed a marked improvement, with collecting tubules lined by normal cuboidal cells showing intact cellular architecture and spherical nuclei, and the absence of interstitial hemorrhage (Fig. 12 d).

#### Immuno-histochemical findings

**Fig. 13:**
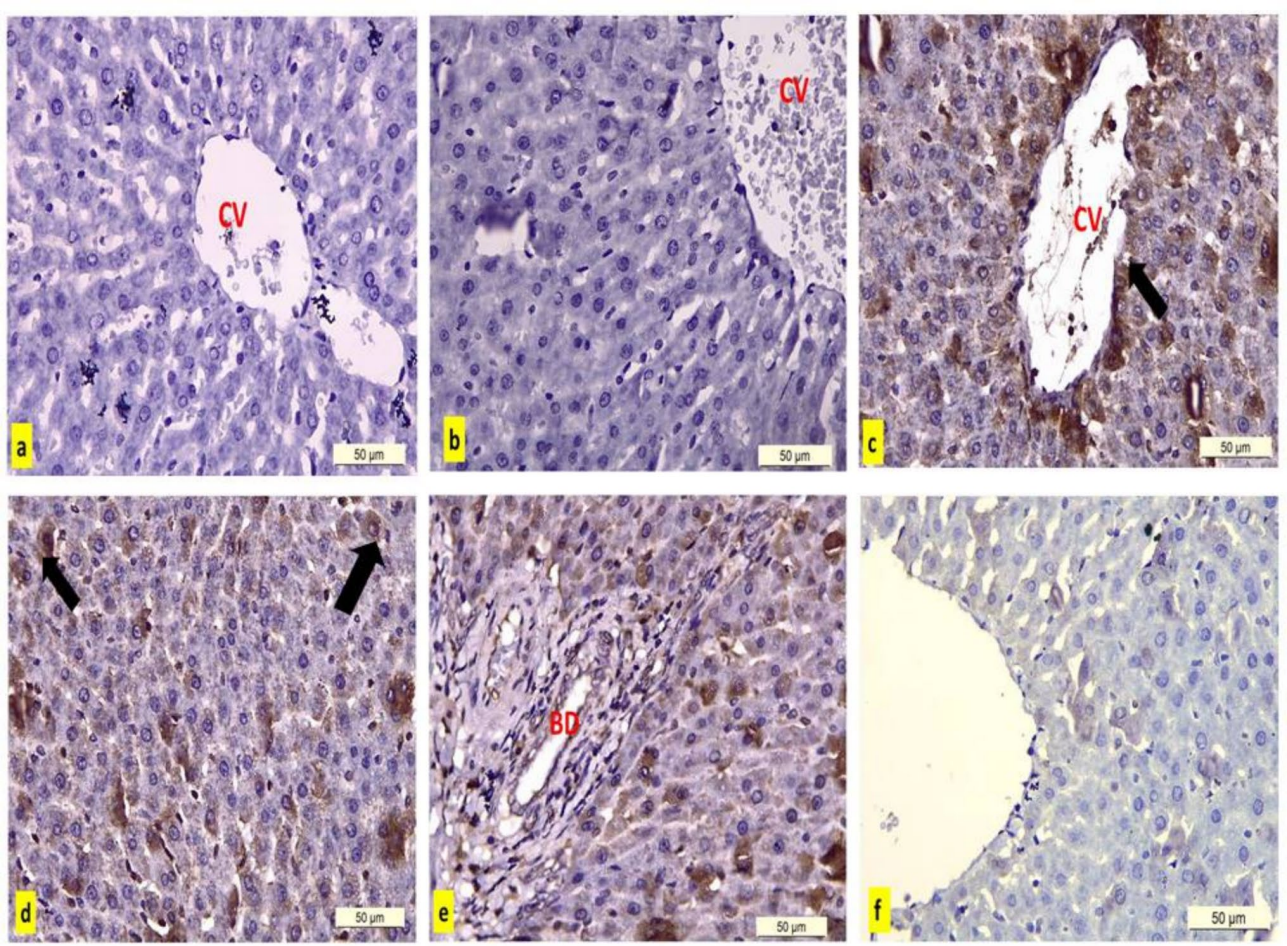
Photomicrographs showing immunoexpression of caspase-3 in liver sections (× 400). **a**, **b** Negative caspase-3 expression in hepatic tissues from the control **a** and CUR-NLs -treated **b** groups. **c**–**e** Intense caspase-3 expression in the DBP-exposed group. **f** Weak caspase-3 expression in the DBP + CUR-NLs co-treated group

Immunohistochemical analysis of liver sections revealed that both the control and CUR-NLs groups showed negative immunoreactivity for caspase-3, indicating the absence of apoptotic activity (Fig. 13 a, b). In contrast, the DBP-intoxicated group exhibited strong caspase-3 immune expression, particularly in hepatocytes (Fig. 13 c–e). However, liver sections from the CUR-NLs-treated group showed only mild caspase-3 immunoreactivity (Fig. 13 f), suggesting that curcumin supplementation attenuated DBP-induced apoptosis.

**Fig. 14:**
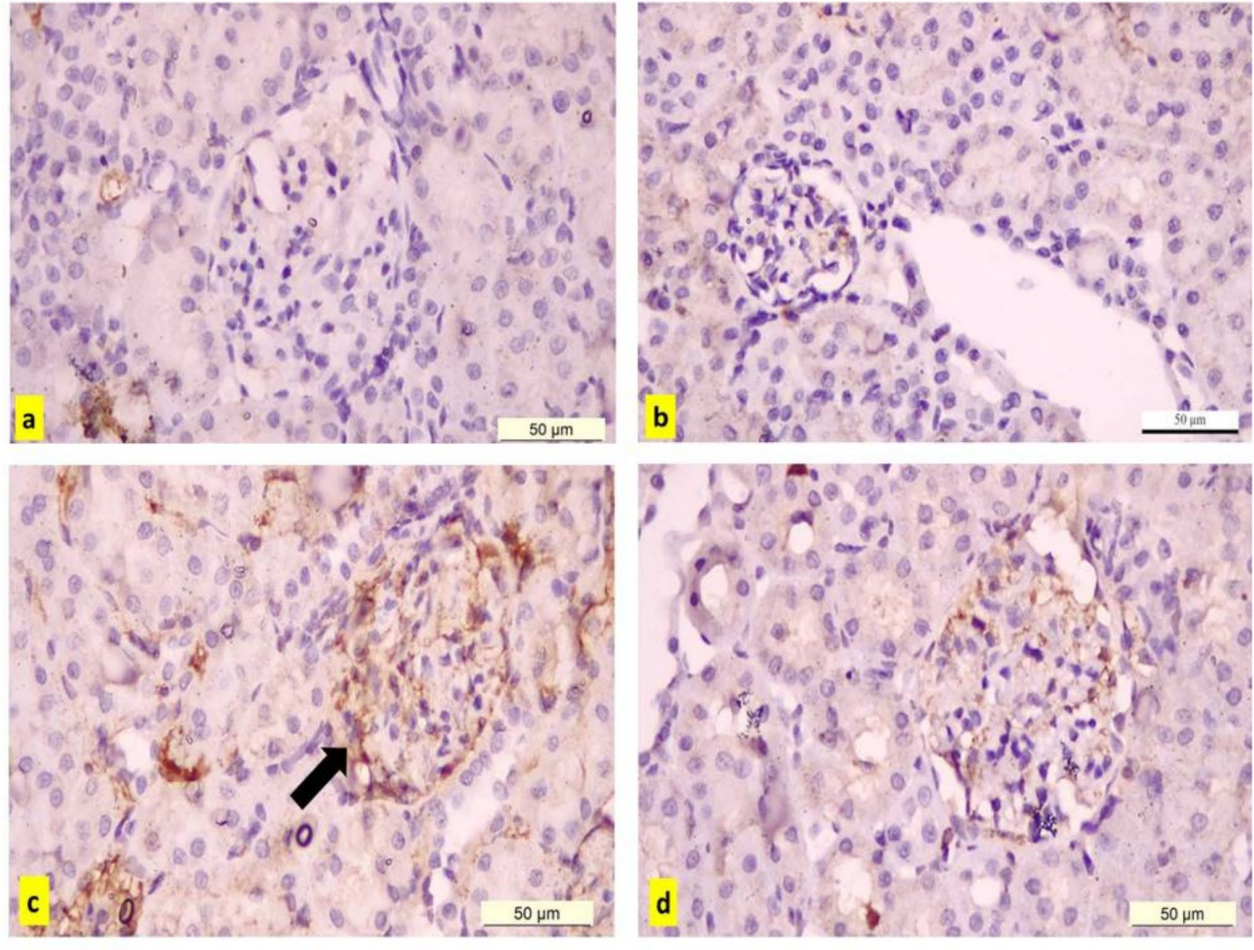
Photomicrographs showing caspase-3 immunoexpression in renal cortex sections (× 400). **a**, **b** Negligible caspase-3 expression in renal tissues from the control **a** and CUR-NLs **b** groups. **c** Strong positive expression in the DBP-treated group. **d** Mild caspase-3 expression in the DBP + CUR-NLs -treated group

**Fig. 15:**
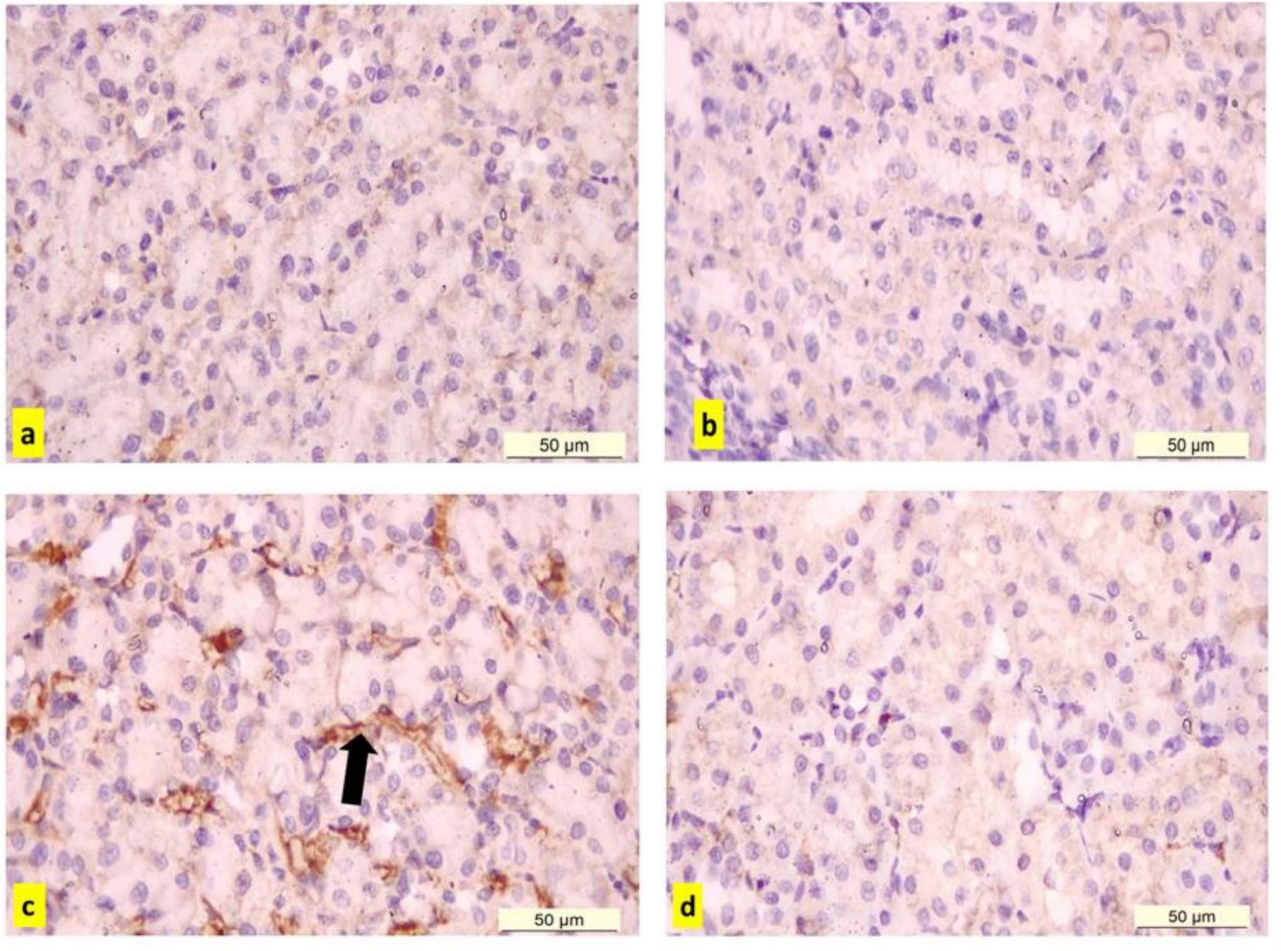
Photomicrographs show caspase-3 expression in renal medulla sections (× 400). **a**, **b** Mild positive caspase-3 expression in renal tissues from the control **a** and CUR-NLs **b** groups. **c** Intense positive expression in the DBP-treated group. **d** Moderate expression in the DBP + CUR-NLs -treated group

Immunohistochemical examination of the renal cortex and medulla in the control and CUR-NLs -only groups showed negligible caspase-3 immunoreactivity (Figs. 14a,b and 15a,b). In contrast, the DBP-intoxicated group exhibited strong positive caspase-3 immunoreactivity (Figs. 14 c and 15 c), indicating marked activation of apoptotic pathways. However, the DBP + CUR-NLs co-treated group displayed moderate immunoreactivity (Figs. 14 d and 15 d), suggesting a partial protective effect of CUR-NLs against DBP-induced apoptosis.

## Discussion

The present work uniquely evaluates the effectiveness of **curcumin-loaded nanoliposomes (CUR-NLs)** as a targeted nano-delivery system to enhance curcumin bioavailability and therapeutic potency.

### Physicochemical properties and stability of CUR-NLs

These results highlight the potential of curcumin-loaded nanoliposomes (CUR-NLs) as an effective nanocarrier system for preventing DBP- induced toxicity. The optimized formulation showed an encapsulation efficiency of 85%, a zeta potential of −32 mV, and an average particle size of about 45 nm. These characteristics indicate excellent physicochemical stability, uniform particle dispersion, and high drug entrapment efficiency, which are key parameters for nanocarriers intended to enhance the solubility, circulation time, and bioavailability of substances like curcumin that are poorly soluble (Chen et al. 2015; Maeso et al. 2024).

Consistent with earlier findings, liposomal encapsulation significantly improved curcumin’s solubility, stability, and resistance to environmental degradation (Chen et al. 2015). Maeso et al. (2024) further proved that curcumin nanoliposomes exhibit improved sustained-release behavior and potent intracellular antioxidant activity, supporting their potential use in the management of oxidative stress–related disorders. In agreement with these studies, our physicochemical stability evaluation showed that particle size and EE% were maintained over 30 days at 4 °C, confirming that CUR-NLs can preserve their structural integrity and bioactivity during short-term storage (Chen et al. 2015).

### In vivo protective efficacy of CUR-NLs

Functionally, our results confirm the therapeutic potential of CUR-NLs. Bakeer et al. (2025c) reported that oral intake of CUR-NLs (100 mg/kg/day for 60 days) significantly ameliorated DBP-induced testicular toxicity by restoring serum testosterone, LH, and FSH levels, and upregulating INSL3 expression. Similarly, oxidative and apoptotic markers (MDA, LDH, caspase-3, and caspase-9) were markedly reduced, suggesting modulation of the PI3K/AKT/mTOR and *Nrf2*/SOD/CAT signaling pathways (Soliman et al. 2025b).

Our current findings in liver and kidney tissues extend this evidence, showing that CUR-NLs effectively modulate redox homeostasis and apoptosis-related gene expression, thereby providing multi-organ protection. The physicochemical stability of CUR-NLs is closely associated with their in vivo efficacy, as enhanced bioavailability and tissue distribution contribute to potent antioxidant and antiapoptotic effects. Collectively, these results support the translational potential of this nano formulation and highlight the need for further pharmacokinetic characterization (e.g., C_max, AUC, and half-life) and comprehensive tissue distribution studies before clinical application.

### DBP-induced hepatorenal toxicity

Our study confirmed that DBP exposure causes significant hepatic and renal impairment, as demonstrated by increased blood levels of ALT, AST, LDH, creatinine, uric acid, and urea. Oxidative stress was apparent from the increased MDA concentration and decreased catalase activity. These results are in line with accumulating evidence that DBP disrupts mitochondrial function, promotes lipid peroxidation, and induces oxidative and inflammatory injury in hepatic and renal tissues (Ivelja et al. 2025; Singh et al. 2025).

Moreover, recent studies have suggested that ferroptosis contributes to DBP-induced organ toxicity, mediated by iron overload and the activation of the HMGB1–TLR4–NF-κB signaling pathway (Zixu et al. 2025). At the molecular level, DBP exposure in our study was associated with downregulation of antioxidant-related genes (***Nrf2***, ***CAT***, ***SOD***). The depressed expression of the antioxidant-related genes confirms the findings of earlier studies, which demonstrated that ROS-induced oxidative stress is the underlying mechanism of DBP-induced damage (Rashad et al. 2018; Bakeer et al. 2025a; Bakeer et al. 2025b). The dysregulation of ROS production can lead to uncontrolled apoptosis, contributing to tissue injury (Abuzaied et al. 2026). This process involves key molecules like cytochrome c, caspases, Bcl-2, Bax, and Bak**.** In the present study, the DBP-treated group exhibited a significant increase in the mRNA expression of the *CYCS* and *BAX* genes, and decrease in *BCL2* gene expression. Similarly, Liang et al. (2021) noted that apoptosis is implicated in the DBP-induced renal disorder. Similarly, Cheng et al. (2019) reported that DBP-induced apoptosis in the liver and kidney of Kunming mice is associated with dysregulation of the extracellular signal-regulated kinase (ERK1/2) pathway.

### Protective mechanisms of CUR-NLs

Our current results confirmed the efficiency of CUR-NLs against DBP-induced hepatorenal injury in terms of modulation of oxidative stress and apoptosis pathways. To the best of our knowledge, this study is among the first to investigate the influence of CUR-NLs against DBP-induced hepato-renal injury. Nanoliposomes represent an effective strategy to enhance drug bioavailability and boost its efficiency (Youssef et al.^2026^; Zhao et al.^2025^). Co-treatment with CUR-NLs significantly restored hepatic and renal functional markers, suppressed lipid peroxidation, normalized antioxidant enzyme activities, and modulated the expression of apoptosis-related genes. These findings are consistent with curcumin’s well-documented ability to activate the Keap1–*Nrf2*/ARE signaling pathway, thereby enhancing the expression of antioxidant enzymes such as SOD, CAT, GPx, and HO-1, while concurrently inhibiting the NF-κB and MAPK signaling pathways to attenuate inflammation and apoptosis **(**Cui et al. 2025; Bakeer et al. 2025c). Furthermore, our results demonstrated that CUR-NLs normalized serum protein fractions, including albumin and globulins, which serve as sensitive indicators of hepatic biosynthetic function and effect on reproduction (Soliman et al. 2024; Bakeer & Hendawy 2025). The restoration of protein metabolism further suggests that CUR-NLs preserve hepatocellular integrity and maintain systemic protein homeostasis, in agreement with previous reports demonstrating its hepatoprotective potential (Liang et al. 2021; Suhair et al. 2024).

Histopathological evaluation revealed pronounced DBP-induced structural damage in both hepatic and renal tissues, characterized by sinusoidal congestion, hepatocellular vacuolation, inflammatory cell infiltration, glomerular shrinkage, and tubular degeneration, consistent with previous studies (Cheng et al. 2019; Anis et al. 2023). Immunohistochemical detection of caspase-3 confirmed apoptosis as a major mechanism underlying DBP-induced cytotoxicity. Notably, treatment with CUR-NL significantly alleviated these pathological changes, preserving the normal hepatic and renal architecture and markedly reducing caspase-3 immunoexpression. Collectively, these findings substantiate the potent antioxidant and antiapoptotic properties of CUR-NLs in protecting against DBP-induced hepatorenal injury (Samarghandian et al. 2017).

### Translational significance and future directions

Phthalates, including di-n-butyl phthalate (DBP), are pervasive environmental toxicants, with increasing evidence linking their exposure to reproductive, hepatic, and renal dysfunction (Ivelja et al. 2025). Identifying safe dietary or nutraceutical interventions to mitigate such toxicity holds significant translational relevance for health. Curcumin-loaded nanoliposomes (CUR-NLs) offer a promising platform that combines curcumin’s pharmacological activity with enhanced solubility, stability, and bioavailability. Recent research focuses on natural antioxidants **(**Ahmed et al. 2025;Soliman and Bakeer 2026; Khormi et al. 2026; El-Gendy et al., 2025). Future investigations should include comprehensive pharmacokinetic profiling, biodistribution analysis, and long-term safety evaluation of CUR-NLs in both preclinical and clinical settings. Further exploration of additional target organs and deeper mechanistic studies of antioxidants (*Nrf2*/Keap1) and apoptotic (Bcl-2 family, caspases) signaling pathways will help to establish the full therapeutic potential of CUR-NLs in mitigating DBP-induced toxicity.

## Conclusion

This research shows that DBP exposure causes marked hepatic and renal injury in rats via oxidative stress and apoptosis-related mechanisms. Curcumin-loaded nanoliposomes (CUR-NLs) effectively mitigated these alterations by enhancing antioxidant enzyme activities, decreasing lipid peroxidation, improving protein metabolic function, and modulating the expression of apoptosis-associated genes. Histopathological and immunohistochemical analyses further corroborated the protective efficacy of CUR-NLs. These results highlight the novelty of nanoliposome-encapsulated curcumin as an effective therapeutic approach for counteracting DBP-induced hepatorenal toxicity. Future investigations should emphasize pharmacokinetic characterization, tissue distribution, and long-term safety assessments to facilitate the translational application of CUR-NLs in the prevention of phthalate-induced organ toxicity. These findings suggest that CUR-NLs may represent a promising preclinical candidate for further investigation.

## Data Availability

All source data for this work (or generated in this study) are available upon reasonable request.
